# Antagonism of 5‐HT7 Receptors as a Promising Target for Gastric Cancer via Apoptotic Pathway

**DOI:** 10.1002/jbt.70326

**Published:** 2025-06-09

**Authors:** Irfan Cinar, Busra Dincer, Elif Cadirci, Salih Kara, Mehmet Ilhan Yildirgan, Zekai Halici, Saziye Sezin Palabiyik‐Yucelik

**Affiliations:** ^1^ Department of Pharmacology, Faculty of Medicine Kastamonu University Kastamonu Türkiye; ^2^ Department of Pharmacology, Faculty of Pharmacy Ondokuz Mayıs University Samsun Türkiye; ^3^ Department of Pharmacology, Faculty of Medicine Ataturk University Erzurum Türkiye; ^4^ Department of General Surgery, Faculty of Medicine Ataturk University Erzurum Türkiye; ^5^ Department of Toxicology, Faculty of Pharmacy Ondokuz Mayıs University Samsun Türkiye

**Keywords:** 5‐HT7 receptors, caspase, KATO‐III cells, LP44, SB‐269970

## Abstract

Although current treatment strategies have improved clinical outcomes for gastric cancer, they present a challenging prognosis that necessitates novel therapeutic approaches. The 5‐HT7 receptor, a member of the serotonin receptor family, plays a significant role in influencing the pathogenesis of various cancer types. This study seeks to investigate the complex interactions among 5‐HT7 receptors, gastric cancer, and apoptotic processes. A comprehensive set of experimental techniques was employed, including in vitro staining assays for apoptosis assessment, real‐time PCR, and cell proliferation assays. The findings indicate that the 5‐HT7 receptor agonist enhances the proliferation of primary gastric tissue cancer cells and KATO‐III cells, whereas treatment with the 5‐HT7 receptor antagonist significantly inhibits cellular proliferation. Analysis of 5‐HT7 receptor mRNA expression in gastric cancer patient populations indicated significantly elevated levels in cancerous tissues when compared to those in healthy tissues. The administration of a 5‐HT7 receptor agonist (LP44) resulted in increased cell proliferation in primary gastric cancer cells and KATO‐III cell lines, whereas treatment with a 5‐HT7 receptor antagonist (SB‐269970) significantly inhibited proliferation. Additionally, KATO‐III cells treated with the 5‐HT7 receptor antagonist demonstrated a marked upregulation of caspase‐3, caspase‐9, and BAX gene mRNA levels. In contrast, treatment with the 5‐HT7 receptor antagonist was associated with a significant reduction in the expression of nuclear factor kappa B and 5‐HT7 receptor mRNA levels. Annexin V‐FITC/PI and Hoechst 33342 staining demonstrated a pronounced apoptotic effect through antagonism of 5‐HT7 receptors compared to other groups. Collectively, the findings of this study suggest that the enhanced expression of 5‐HT7 receptors influences gastric cancer formation by regulating the apoptotic axis. This provides a novel perspective for understanding the molecular mechanisms underlying the potential of 5‐HT7 receptors as a targeted approach for combating gastric cancer via the apoptotic pathway.

## Graphical abstract

Antagonism of 5‐HT7 receptors as an approach for combating gastric cancer through the apoptotic pathway is targeted. The present study aims to unearth potential avenues for advanced therapeutic strategies in the battle against this devastating disease by exploring the intricate interactions between 5‐HT7 receptors and gastric cancer, as well as apoptotic processes.

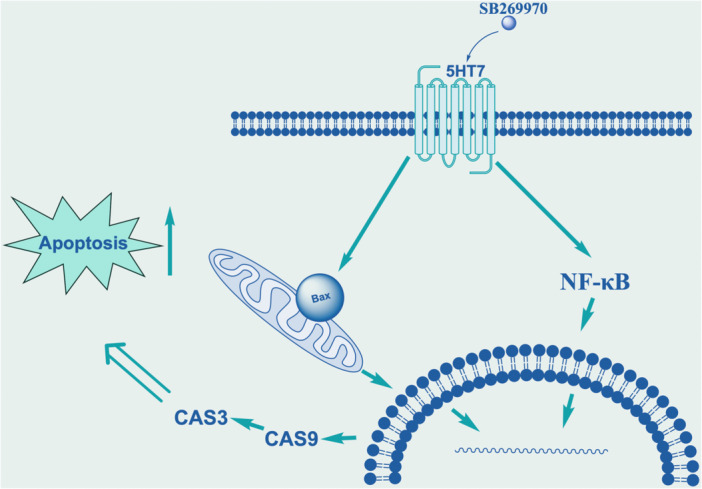

## 1 Introduction

Gastric cancer (GC) stands as a formidable global health challenge, and one of the leading causes of cancer death worldwide [1]. GC is also the highest cancer burden when assessed for disability for patients and the healthcare industry. Approximately 90% of GCs are adenocarcinomas [2]. The clinical outcome for patients with advanced GC is generally poor, despite advances in diagnosis, the development of new chemotherapy protocols, and significant advances in surgical techniques, with 5%–20% 5‐year survival [3].

Traditionally recognized as a neurotransmitter within the central nervous system, serotonin (5‐hydroxytryptamine, 5‐HT) has recently unveiled its diverse effects on cellular processes, including proliferation, apoptosis, and inflammation, within peripheral tissues. Mainly, 5‐HT has been associated with CNS functions. However, its contribution to peripheral diseases such as inflammation [4], prostate cancer [5], lung injury [6], dumping syndrome [7], and colitis [8] has also been shown. In addition to the properties of 5‐HT, it is a mitogenic factor for various reasons. Since 5‐HT functions as a mitogenic agent in different normal and tumoral cells, 5‐HT receptors may also be involved in the pathophysiology of numerous cancers [9, 10, 11]. This revelation has ignited a surge of interest in understanding the intricate interplay between serotonin and its receptors, particularly the 5‐HT7 receptor, in the context of cancer biology.

The 5‐HT7 receptor, a prominent member of the serotonin receptor family, is associated with numerous disease states and disorders in the central nervous system and periphery [12, 13]. Notably, the expression of the 5‐HT7 receptor has been detected in various cancer tissues, suggesting its potential involvement in the pathogenesis of specific cancer types [14, 15, 16, 17, 18]. In particular, investigations have highlighted the mitogenic properties of serotonin, fueling the proliferation of tumor cells in prostate, pancreatic, lung, and colon cancer. Concurrently, intriguingly, antagonists of the 5‐HT7 receptor have exhibited inhibitory effects on these tumor cells, presenting the 5‐HT7 receptor as a potential target for novel therapeutic interventions. Ectopic serotonin receptor 5‐HT7 was evaluated in adrenocortical carcinoma, and the effects of serotonin on the secretory activities of malignant adrenocortical tumors have been demonstrated [19]. The existence of functioning 5‐HT7 receptors in human glioblastoma cell lines indicates the potential involvement of these receptors in the initiation and progression of cancer [5, 20]. Since serotonin has been recognized as a tumor marker of gastrointestinal carcinoids, as well as, to a certain extent, bronchial, hepatic, and ovarian carcinoids, it is probable that the 5‐HT7 receptor plays a significant role in the pathology of various types of GC [15, 21].

This article examines the potential of 5‐HT7 receptors as a targeted approach for addressing GC through the apoptotic pathway. By examining the intricate interactions between 5‐HT7 receptors, GC, and apoptotic processes, we aim to identify potential avenues for the development of advanced therapeutic strategies in the fight against this devastating disease.

## 2 Methods

### 2.1 Ethical Consent

All phases of the investigations were found to comply with ethical standards by the Ataturk University (Erzurum/Türkiye), Faculty of Medicine, Noninvasive Clinic Research Ethical Committee (20.03.2014; 4/11).

This study included 12 males/females aged from 18 to 75 years old suffering from GC of III or IV degree (common clinical parameter estimated through the cancer staging system).

The criteria for inclusion in the study are as follows: Patients must have a histologically confirmed diagnosis of advanced gastric adenocarcinoma (Stage III/IV) and must have had no chemotherapy, immunotherapy, or targeted therapy 1 month before the study.

### 2.2 Total Rna Purification From FFPE Tissue Sections

Paraffin blocks of 12 male/female patients who received GC diagnosis between 2014 and 2015 at Ataturk University Hospital, Pathology Department (Erzurum/Türkiye), were investigated. Four or five 5‐m sections from each preservation block were freshly sliced to recover the RNA from the FFPE tissue. Only tumors or peritumoral stromal tissues were included after each segment was dissected using a blade. Paraffin was eliminated by using xylene extraction, followed by ethanol washing. Following the manufacturer's instructions, RNA was obtained using the All Prep RNA FFPE kit from Qiagen in Hille, Germany. The concentration of total RNA was examined with the help of the Epoch Spectrophotometer System and Take 3 Plate.

### 2.3 Fresh Samples

12 fresh frozen GC and comparable normal gastric mucosa tissue samples (greater than 10 cm away from the edge of the GC) were taken from individuals with GC within 30 min of resection. These samples were subsequently snap‐frozen in liquid nitrogen and kept at 80°C until use. GC tissue was retrieved from the archived files of the Department of General Surgery at Ataturk University Hospital between January 2015 and December 2016. Two leading pathologists from the hospital's Department of Pathology conducted the histological evaluation of all tissue samples. Before the surgical procedures, informed consent was obtained from each patient.

### 2.4 Primary Cell Culture

All phases of the investigations were found to comply with ethical standards by the National Ethical Committee ‐Ataturk University Medical Experimental Application and Research Center (05.05.2017; 2/03). The gastric adenocarcinoma fragments obtained by surgery were quickly transferred to a cell culture laboratory in Dulbecco's modified eagle's medium (DMEM 4,5 g/dL glucose) that contains 10% fetal bovine serum (FBS) antibiotics. The adenocarcinoma tumor fragments were cut into tiny pieces using sterile scissors. After being carefully separated by centrifugation and washing with phosphate‐buffered saline (PBS), the tumor cells were cultured in a sterile culture flask measuring 25 cm^2^ that contained DMEM at 37°C in a humidified atmosphere of 5% CO_2_. Subconfluent cultures were detached with Trypsin‐EDTA and counted with the trypan blue (Sigma‐Aldrich, USA) counting method. Trypsinization is employed to isolate tumor cells when there is a noticeable increase in tumor cells. Inverted microscopes were utilized to observe the cell cultures for roughly 12–72 h.

To determine the IC_50_ value for LP44 and SB‐269970, 5000 cells/well were seeded in a standard medium; after 24 h, the cells were treated with 10, 1, 0.1, 0.01, 0.001 µM LP44, and SB‐269970 in 96‐well plates and incubated for 24 h. After incubation, MTT (5 mg/mL) solution was added and incubated for 4 h. After the required incubation, the cells were solubilized in DMSO to dissolve formazan, the MTT conversion product. Cell optical density absorbance was measured at 570 nm. Drugs were investigated for their activity as single agents. Growth inhibition was expressed as % control (media alone and no drugs) and quantitated by IC_50_ values. All experiments were performed independently at least three times [5].

LP44 is the serotonin 5‐HT7 receptor agonist (Cas no: 824958‐12‐5, Sigma‐Aldrich). SB‐269970 is the serotonin 5‐HT7 receptor antagonist (Cas no: 261901‐57‐9, Sigma‐Aldrich).

### 2.5 KATO‐III Cell Culture

KATO‐III, (Human, Stomach, Gastric Carcinoma) cell line American Type Culture Collection (ATCC, USA) was provided. The cell lines in Cryotube in the liquid nitrogen were removed from the tank and allowed to dissolve in the water bath at 37°C for a short time. Soluble cells were transferred to flasks with T75 cm^2^. After 48 h, KATO‐III cells were counted at 2 × 10^5^ cells/well in DMEM containing 10% FBS and, seeded in a 96‐well plate and incubated at 37°C in a humid atmosphere containing 5% CO_2_.

### 2.6 Real‐Time Monitoring of Cell Growth and Proliferation Assay

The xCelligence system, a label‐free method for dynamic monitoring of living cells, was used to evaluate the growth of primary GC and KATO‐III cells. All substances were serially diluted, and cells were subjected to a final DMSO concentration of 0.1%. Control cells are also exposed to 0.1% DMSO. First, DMEM media was used to seed 5000 primary GC or KATO‐III cells into each well. After 24 h, the cells were treated with 10^‐6 ^M LP44 and SB‐269970. Plates with gold electrodes were used in the xCELLigence system. The Cell index, the interaction of cells and electrodes, reflects cell number, adhesion, and growth and is measured as an impedance response. During the experiment, automatic measurements were made every 15 min. The data are accessible as a normalized cell index.

### 2.7 Real‐Time PCR

The procedures for total RNA extraction and cDNA synthesis followed those detailed in our earlier work [22]. MRNA was extracted from the homogenized KATO‐III cells. Following the manufacturer's recommendations, total mRNA was purified using the QIACUBE apparatus. The High‐Capacity cDNA Reverse Transcription Kit was used to reverse‐transcribe RNA samples into cDNA [10]. We use a spectrophotometer to determine the purity and quantity of the mRNAs and cDNAs we have obtained.

### 2.8 Relative Quantification of Gene Expression

Relative 5‐HTR7, NF‐kB, CAS‐3, CAS‐9, and BAX mRNA expression analyses were performed with the StepOne Plus real‐time polymerase chain reaction (PCR) system using cDNA synthesized from KATO‐III cells and 5‐HTR7 mRNA expression level paraffin block samples and fresh gastric sample's RNAs. As shown in Table 1, primers designed for humans were used in real‐time PCR.

**TABLE 1:** Primary sequences used in real‐time PCR.

Gene	Human	Forward (5′‐3′)	Reverse (5′‐3′)
5‐HTR7	Primer Design	TGGTGATCTCCGTGTGCTTCGT	AGGGTGGTGGCTGCTTTCTGTT
CAS‐3	Primer Design	TGTAGAAATGATGATGTGGAAGAAC	GCAGTTAAGTCATCCGTGTATATC
CAS‐9	Primer Design	CCAGTGACAGACAGGCTCTTA	GCAATCCACGGCATTCATCT
BAX	Primer Design	ATGGAGCTGCAGAGGATGAT	CAGTTGAAGTTGCCGTCAGA
NF‐kB 1	Primer Design	GTAACTGCTGGACCCAAGGA	CCTCTGTCATTCGTGCTTCC
β‐Actin	Primer Design	GCAAGCAGGAGTATGACGAGT	CAAGAAAGGGTGTAACGCAACTAA

Results were compared to the control groups and shown as relative fold. Endogenous controls (ß‐actin) were used as expression data for each cell group. Compared to our past investigations, the study was conducted per standard procedure and in line with the manufacturer's instructions [23]. All results were reported as fold changes in expression compared to the cell groups using the 2^‐ΔΔ Ct^ technique [24].

### 2.9 Hoechst 33342 Staining Assay

Hoechst 33342 staining was used to identify apoptotic cell death. The investigation was carried out according to the guidelines provided by the manufacturer and our prior research. Modifications were tracked using a LEICA inverted microscope [5].

### 2.10 Annexin V‐FITC/PI Double Staining Assay

KATO‐III cells (2.5 × 10^5^/well) were seeded in 6‐well plates, and 5‐HT7 receptor agonist/antagonist were applied for 24 h. Cells were obtained using the AnnexinV‐FITC detection kit according to manufacturer instructions. CytoFLEX identified the red fluorescence from PI and the green fluorescence from Annexin V‐FITC. CytExpert Software used the data to analyze [5].

### 2.11 Statistical Analysis

The cell culture studies were performed three times, and the data were given as mean ± standard deviation (SD). The Shapiro‐Wilk test was employed to ascertain compliance with a normal distribution (Gaussian) for each group. A p‐value exceeding 0.05 was deemed indicative of a normal distribution. Furthermore, the Skewness and Kurtosis tests were employed to ascertain the normality of the distribution, while Levene's test was utilized to assess the homogeneity of variances. ANOVA was used to analyze variance to find the significant differences between the groups. Tukey posttest was utilized for several comparisons. Statistics were considered significant for *p‐values* less than 0.05. When the normality distribution of the data was rejected, comparisons between the two groups were performed using the Mann‐Whitney U test. The Whitney U test was used to determine whether there were any 5‐HT7 receptor differences between normal and malignant tissues. Similar tests were conducted on Windows using GraphPad Prism 6.0.

## 3 Results

### 3.1 Upregulation of 5‐HT7 Receptor in GC Tissues

**FIGURE 1:**
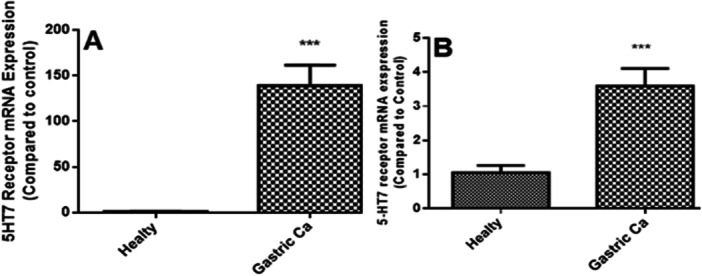
Comparison of 5‐HT7 receptor mRNA expression obtained from GC FFPE tissues with healthy tissues (A). 5‐HT7 mRNA receptor expression in healthy and cancerous fresh tissues from patients with GC (B). The results were evaluated according to the Mann‐Whitney U test. ***p < 0.001 was significant.

Our study first looked at 5‐HT7 receptor expression levels from GC FFPE tissues and compared them with healthy FFPE tissues. Patients’ files were also reviewed at this stage, and real‐time PCR data were compared. All the patients had adenocarcinomas; some tumors were T4N2, and others were T4N3. In our FFPE tissue analysis, the expression of 5‐HT7 receptor mRNA detected in cancer tissues increased significantly compared to healthy tissues. Here, the importance of the 5‐HT7 receptor in GC tissue is seen (Figure 1A). The same comparisons were made in fresh tissues taken from surgeries to validate results. Here, the 5‐HT7 mRNA expression level in GC was significantly increased compared to the control group (*p* < 0.001) (Figure 1B).

### 3.2 Reducing GC Cell Proliferation via 5‐HT7 Receptor Antagonist

**FIGURE 2:**
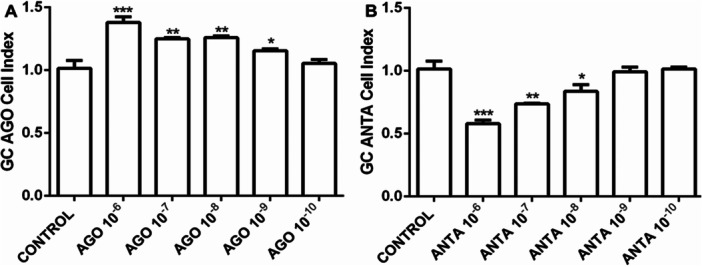
The effect of different doses of the 5‐HT7 receptors agonist (LP44) and antagonist (SB‐269970) on dose‐dependent cell proliferation in the GC cell line in 72 h. (A) agonist (LP44). (B) antagonist (SB‐269970). *p < 0.05, **p < 0.01, and ***p < 0.001 indicate levels of significance compared to the control group.

The IC_50_ for LP44 and SB‐269970 were determined in the cell line tested. For the primary GC cell line, the agonist and antagonist IC_50_ values were 1.09 and 8.84 µM, respectively. LP44 was stimulating and SB269970 was inhibiting cell proliferation significantly at 10^‐6 ^M concentrations (Figure 2A, B).

**FIGURE 3:**
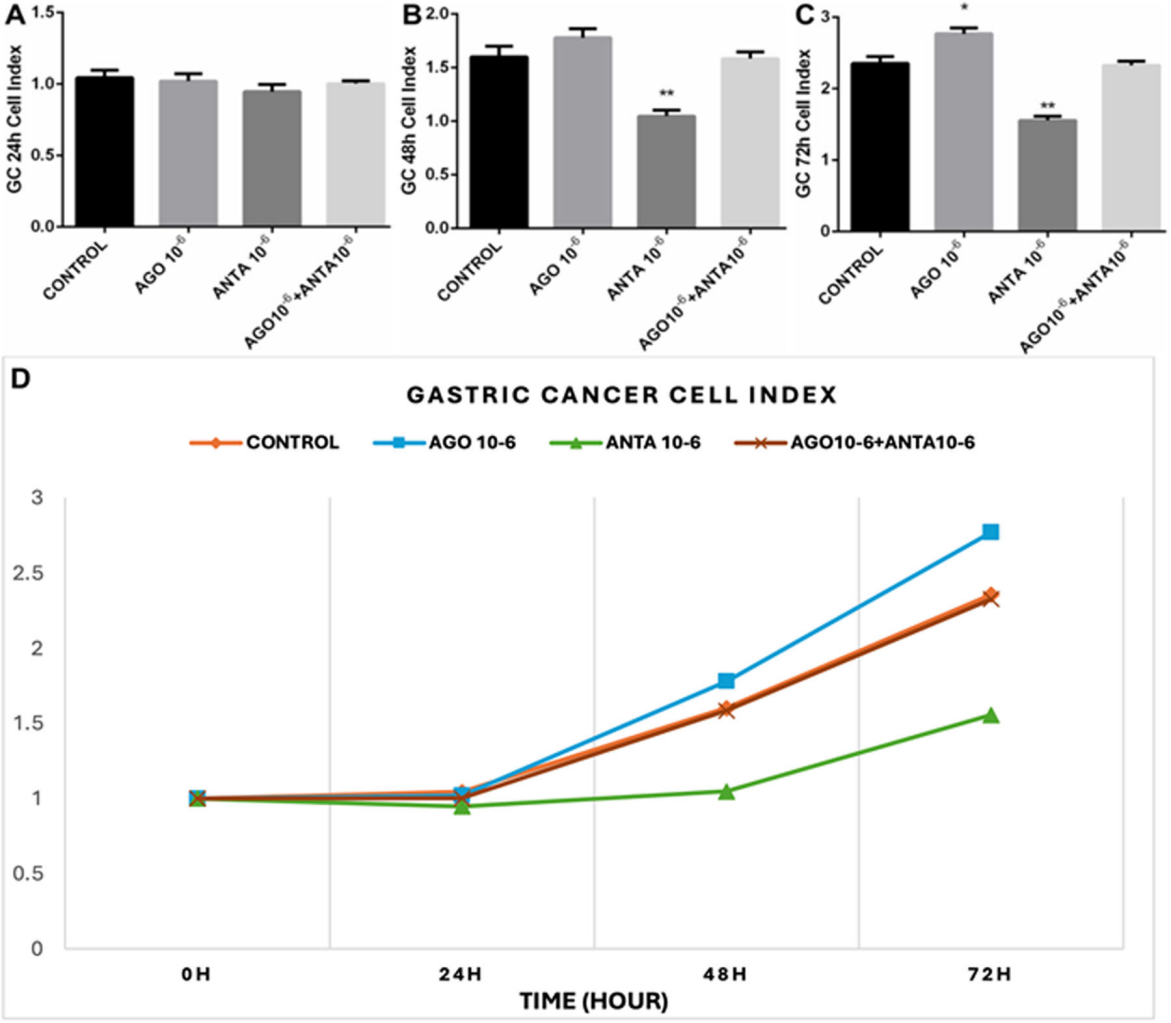
Effect of 10⁻⁶ M doses of the 5‐HT7 receptor agonist (LP44) and antagonist (SB‐269970) on time‐dependent cell proliferation in the GC cell line at 24 (A), 48 (B), and 72 (C) hours, along with the control group and time‐dependent cell index (D). The results were evaluated according to the Tukey test. *p < 0.05, **p < 0.01, ***p < 0.001 indicate levels of significance compared to the control group.

Based on this result, we performed a time‐dependent viability test with LP44 and SB‐269970 doses that we applied to GC cells via the xCELLigence. 5‐HT7 receptor antagonist administration significantly inhibited GC cells in a time‐dependent manner (Figure 3A–D).

**FIGURE 4:**
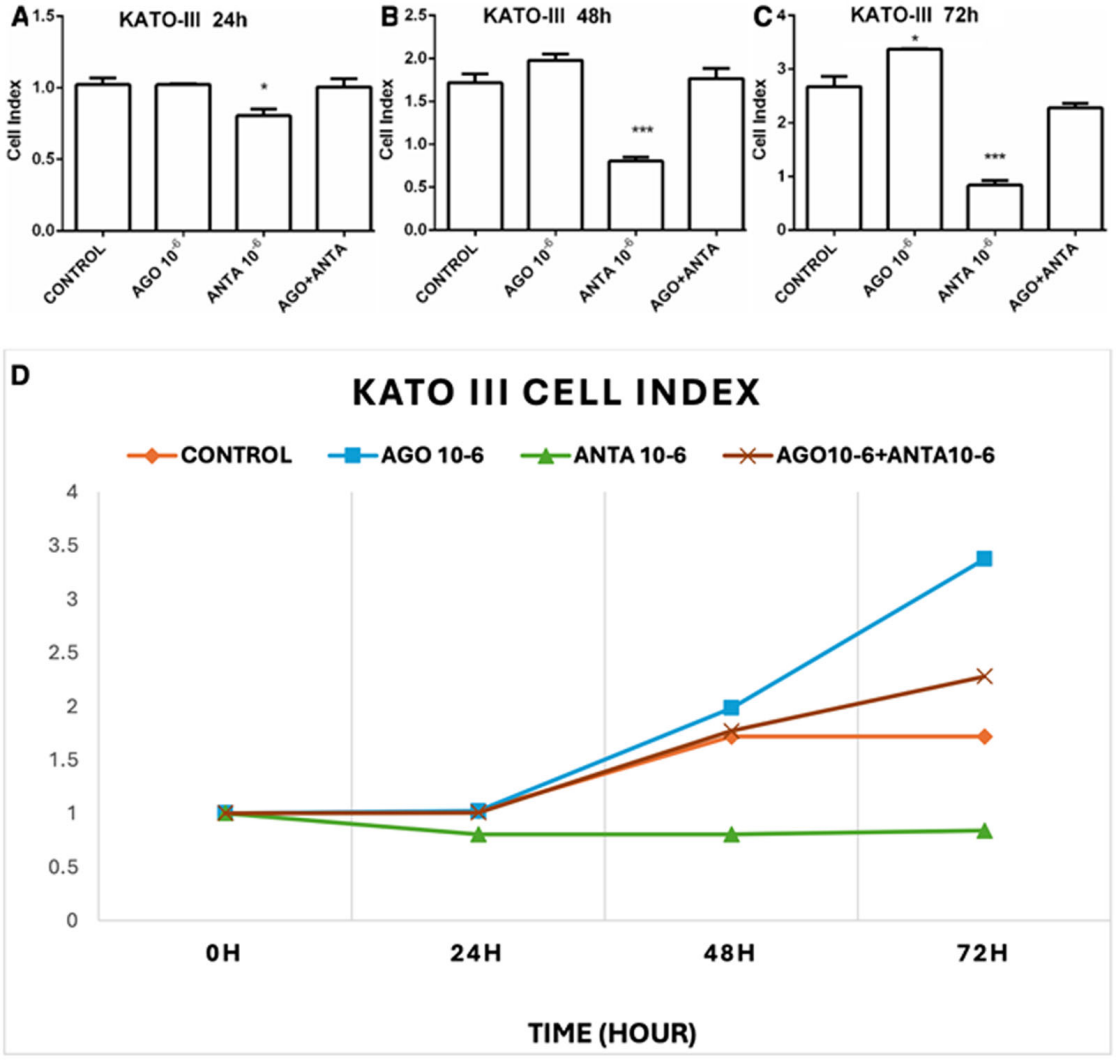
Effect of 10⁻⁶ M doses of the 5‐HT7 receptor agonist (LP44) and antagonist (SB‐269970) on time‐dependent cell proliferation in the KATO‐III cell line at 24 (A), 48 (B), and 72 (C) hours, along with the control group and time‐dependent cell index (D). The results were evaluated according to the Tukey test. *p < 0.05, **p < 0.01, ***p < 0.001 indicate levels of significance compared to the control group.

Similarly, our application was made for the KATO‐III cell line. The effects of the 5‐HT7 receptor agonist and antagonist on proliferation were followed by xCELLigence. Again, the administration of the 5‐HT7 receptor agonist did not alter cell proliferation at 24 (Figure 4A) and 48 (Figure 4B) hours but increased it at 72 (Figure 4C) hours. The 5‐HT7 receptor antagonist administration significantly inhibited KATO‐III cells in a time‐dependent manner (Figure 4D).

### 3.3 Antagonism of the 5‐HT7 Receptor Ameliorates GC by Affecting Apoptotic Gene Regulation

**FIGURE 5:**
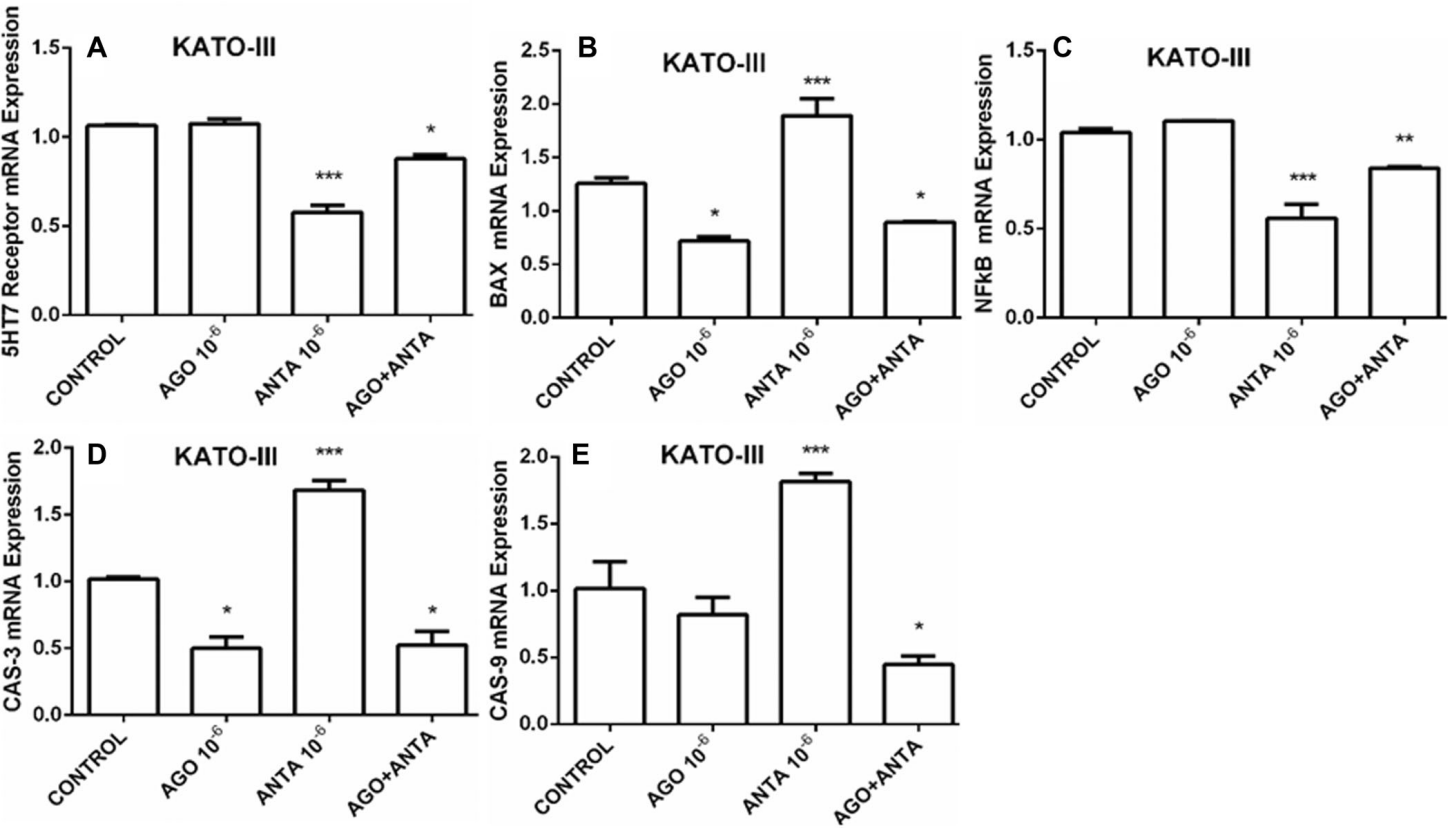
5‐HTR7 (A), BAX (B), NF‐kB (C), CAS‐3 (D), and CAS‐9 (E) mRNA expression analyses from KATO‐III cell. The results were evaluated according to the Tukey test. *p < 0.05, **p < 0.01, ***p < 0.001 indicate levels of significance compared to the control group.

We observed that agonist administration decreased 5‐HT7 (Figure 5A) receptor expression and increased antagonist application in the investigations in KATO‐III cells. In addition, NF‐kB (Figure 5C) mRNA expression, which contributed to cancer development, was no significant change with agonist administration but decreased with antagonist administration. Improving the body's anticancer defense mechanisms, BAX (Figure 5B), CAS‐3 (Figure 5D), and CAS‐9 (Figure 5). mRNA expression by an antagonist supports the possible anticancer effect of 5‐HT7 receptors with antagonists (Figure 5E).

### 3.4 5‐HT7 Receptor Antagonist Reduces Cell Viability and Repaired Impaired Apoptosis in KATO‐III Cell Line

**FIGURE 6:**
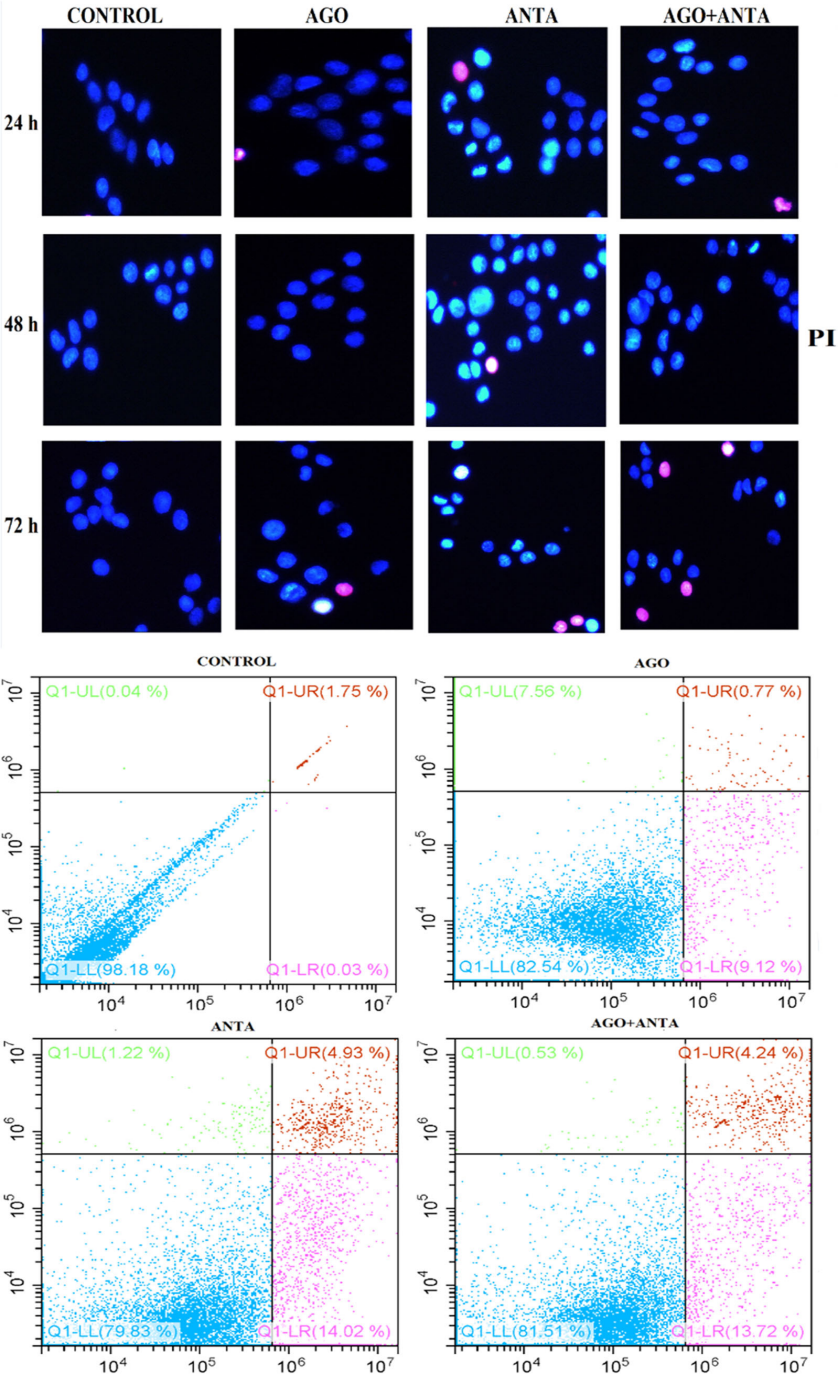
Fluorescent microscope findings after Hoechst 33342 staining in the KATO‐III GC cells (A). KATO‐III 24‐h Annexin V/PI Apoptosis Levels (B). AGO: LP44 (10⁻⁶ M), ANTA: SB‐269970 (10⁻⁶ M).

Hoechst 33258 staining was first used to demonstrate apoptosis in KATO‐III cells. When examined under a fluorescent microscope, significant apoptosis with chromatin agglutination and karyopyknosis was observed in the ANTA group (Figure 6A). In the control group, the cells and their nuclei were normal. The Annexin V/PI double labeling test was conducted and analyzed by flow cytometry to conclusively demonstrate that the cell death brought on by the 5‐HT7 receptor antagonist is apoptosis. As shown in Figure 6B, the ANTA groups had a more significant percentage of apoptotic cells than LP‐44. Although apoptotic cells were present in the AGO + ANTA groups, this apoptotic rate was lower than that of the ANTA group. These results supported that the 5‐HT7 receptor antagonist could induce apoptosis in the KATO‐III cells (Figure 6B).

### 3.5 GC Patients’ Immunohistopathological Result

**FIGURE 7:**
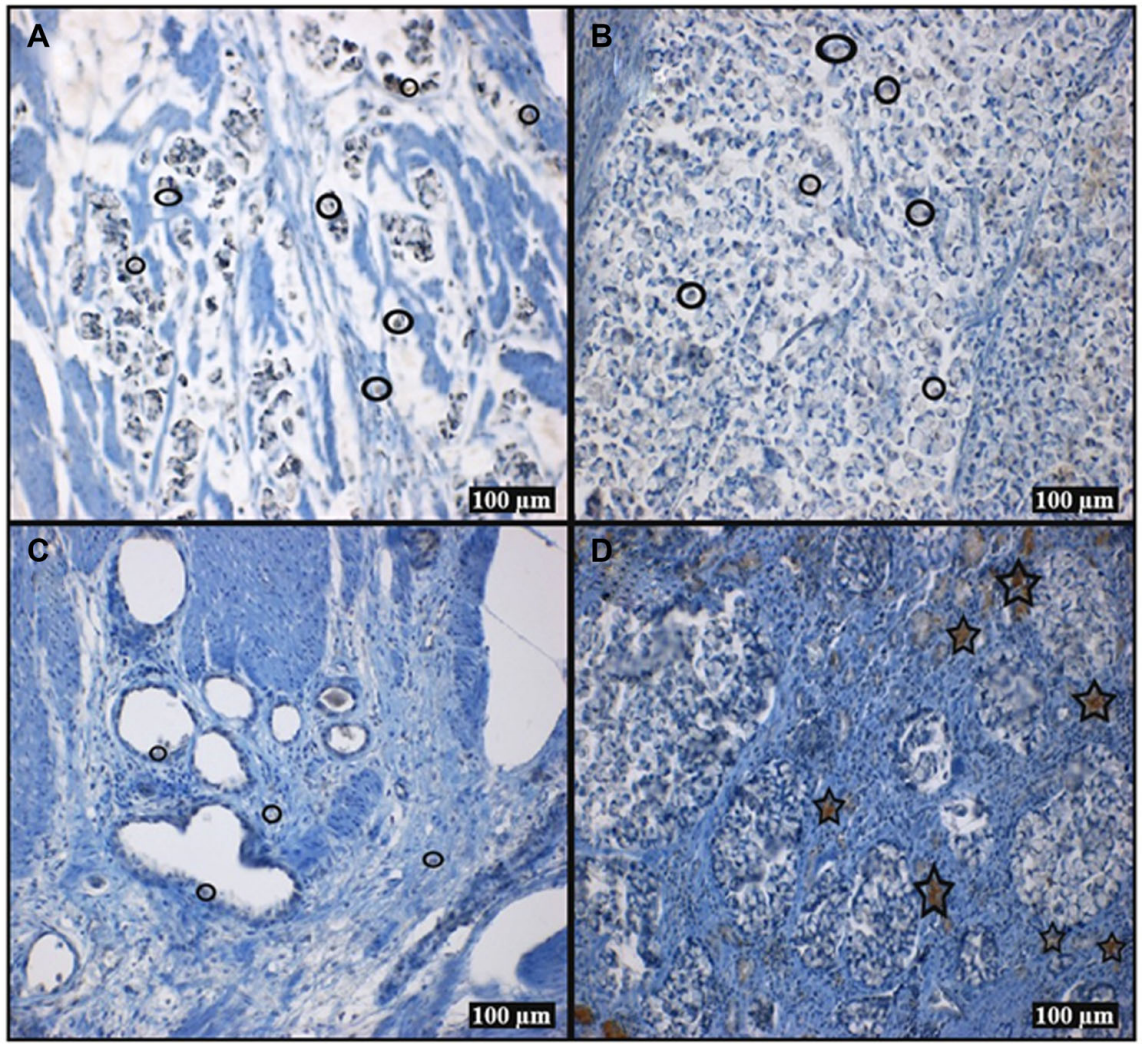
Immunohistochemistry result of the 5‐HT7 receptor Ab dyed stomach sections with tumor cells (A–C) and inflammatory cells (D) (Star: strength immune staining, round: weak immune staining).

In the immunohistochemical staining results with the 5‐HT7 receptor antibody, staining at different rates was observed in selected FFPE tissue samples. According to the dyeing results, the strength and intensity of staining in tumor cells are weak (Figure 7A–C). There is no staining in tumor cells, while adjacent inflammatory cells show staining (Figure 7D).

## 4 Discussion

The pathogenesis and progression of GC involve intricate interactions of multiple factors, rendering it a complex process. Currently, GC stands as one of the most prevalent malignant tumors, posing a significant global health burden. Despite advancements in early detection and standard treatment approaches, the mortality rates associated with GC remain alarmingly high [25]. One of the challenges in diagnosing GC lines is its diverse and nonspecific gastrointestinal symptoms, which can easily mimic benign conditions like gastroesophageal reflux or peptic ulcer disease, potentially leading to missed diagnoses. Therefore, a comprehensive medical history should be meticulously obtained when evaluating patients suspected of GC and presenting possible risk factors such as unexplained weight loss, bleeding, epigastric burning, loss of appetite, vomiting, pain, or discomfort. This should be supplemented by a thorough physical examination and, particularly, endoscopy, considering social factors as well [26].

While patients diagnosed with serotonin‐producing carcinoid malignant tumors in the jejunum, ileum, and cecum typically have a favorable prognosis with long survival expectancy, tumor progression may manifest more rapidly in certain cases [27]. Intriguingly, research has revealed that serotonin elevation in various tumor cells, including prostate, pancreatic, lung, and colon cancer, stimulates tumor growth [28], while antagonists demonstrate inhibitory effects on these cells [29]. Consequently, pharmacotherapy targeting serotonin receptors has become a potential therapeutic avenue. Despite this, limited data have been available on the involvement of serotonin in cancer cell migration and metastatic processes. Although direct pathways of serotonin regulation and tumor proliferation have only been established in certain carcinoids [30, 31], serotonin has been identified as a tumor marker for gastrointestinal carcinoids and, to some extent, bronchial, hepatic, and ovarian carcinoids [32]. Furthermore, therapeutic strategies involving the inhibition of serotonin transporter and modulation of serotonin synthesis using selective serotonin reuptake inhibitors (SSRIs) have also been explored.

Predominantly situated in the thalamus and hypothalamus, the 5‐HT7 receptor stands as one of the more recently identified members within the serotonin receptor family [33, 34]. Although its functions are not yet fully elucidated, its presence has also been observed in the periphery, specifically in smooth muscle cells of blood vessels [33, 35], as well as in the gastrointestinal tract [33], where it plays a role in regulating [36]. Notably, certain studies have suggested that 5‐HT7 receptor agonists can reduce gastric‐fundus tone, but the impact of 5‐HT7 receptor antagonists on gastric accommodation remains to be explored [37]. This study demonstrates that the 5‐HT7 receptor is associated with proliferation in GC and KATO‐III cell lines. Furthermore, it establishes that proliferation in these cell lines is inhibited by applying a 5‐HT7 receptor antagonist, which impacts the apoptotic pathway.

In addition to being a neurotransmitter, 5‐HT has essential physiological functions as a hormone in the gastrointestinal system [38]. 5‐HT proliferates various tumor cells (carcinoma types, carcinoids, etc.) [39]. 5‐HT has been most prominently associated with carcinoid syndrome, which is named carcinoid tumors by secreting several factors. Although 5‐hydroxyindolacetic acid (5‐HIAA), a breakdown product of 5‐HT that is excreted in the urine, is frequently used as a marker in the diagnosis and follow‐up of patients with carcinoid syndrome, urinary 5‐HIAA excretion has also been linked to the prognosis and severity of carcinoid heart disease in those patients [40]. Our study showed that the levels of 5‐HT7 receptor mRNA expression were significantly higher in the material obtained from FFPE tissues diagnosed with GC as well as in fresh gastric cancerous tissues compared to control, which is good for increasing the serotonin level in tumor tissues. Previous studies have observed the expression of the 5‐HT7 receptor in MDA‐MB‐231 breast cancer cells [39]. Additionally, the expression of the 5‐HT receptor HTR2B was found to be significantly elevated in human gastric adenocarcinoma tissues compared to nontumor tissues [42]. However, the effect of 5‐HT7 on gastric tissues, primary GC cells, and the KATO‐III cell line had never been investigated. In our study, the presence of serotonin 5‐HT7 receptors in gastric tissue, primary GC cell, and KATO‐III cell line, the effect of this receptor agonist and antagonist on these cell lines was investigated as proliferation and mRNA expression levels.

Some cancer cell line (typical (NCIH727), atypical (NCI‐H720) bronchopulmonary NET, small intestine NET (KRJ‐I), and human pancreatic carcinoid cell line (BON)) studies have shown the proliferative effects of serotonin [41, 42]. Current studies investigate the autocrine proliferation role of serotonin in human carcinoid cells 5‐HT1A, 1B receptors in pancreatic tumors and 5‐HT2 receptors in bronchopulmonary NET and small intestine NET [39]. In another study, SB‐26997 was shown to have an antiproliferative effect on breast cancer cell lines [43]. Similarly, in our current study, both 5‐HT7 receptor agonist proliferative effect and 5‐HT7 receptor antagonist showed an inhibitory effect in both primary GC cells and the KATO‐III cell line. There are also several publications reporting a regulatory role of 5‐HT7 in the expression of other proteins and receptors [5, 22, 46]. Both 5‐HT7 receptor agonists and antagonists can affect receptor expression through intracellular signaling cascades. These cascades involve second messengers, protein kinases, and transcription factors that modulate gene expression. Thus, receptors may be upregulated or downregulated in the presence of agonists or antagonists. These regulatory mechanisms allow cells to adjust their sensitivity to external signals and maintain physiological balance in response to varying receptor activation levels [43].

BAX, a member of the Bcl‐2 family, is a multidomain, proapoptotic protein that plays a vital role in the intrinsic apoptotic pathway. Stimulation of death movements causes conformational changes in BAX, which then moves from the cytoplasm to the mitochondria, thus ending the event with mitochondrial apoptosis [44]. The Bcl‐2 protein tightly controls the proapoptotic activity of BAX. The BAX/Bcl‐2 ratio is crucial for triggering intrinsic apoptosis because Bcl‐2 forms heterodimers with BAX and inhibits BAX oligomerization at the mitochondrial outer membrane [45]. Our study showed that BAX mRNA levels were changed with the application of 5‐HT7 receptor antagonist in KATO‐III cells.

NF‐kB, another apoptosis marker in cancer cells, has a role in many events, including cell proliferation, apoptosis, angiogenesis, immune response, cell adhesion, and differentiation [46], and high NF‐kB levels have been reported in these events [47, 48, 49]. Studies have reported that NF‐kB is constitutively activated in gastric carcinoma tissue [50]. In contrast, in another study, Kwon HC et al. showed increased expression of NF‐kB in human GC tissue [51]. Supporting all this information, it has been confirmed that inhibiting NF‐kB can promote apoptosis of GC [52], thyroid cancer [53], breast cancer cells, and other tumor cells [54]. According to our results, the expression level of NF‐kB mRNA decreased in KATO‐III cells with the application of 5‐HT7 receptor antagonist in a similar way as in current studies.

Another essential factor in apoptosis during ontogenesis and homeostasis in multicellular organisms is the CAS‐3 enzyme. Therefore, it is an important and potential drug target in cancer research [55]. Activated CAS‐9 cleaves pro‐CAS‐3 into its active form, which induces cell apoptosis [56]. The SGC7901/ADR GC cell line study stated that the activities of CAS‐3 and CAS‐9 have increased [57]. Our results also showed that an increase in caspase activation was associated with an increase in BAX expression. CAS‐3 and CAS‐9 mRNA expression was significantly increased in KATO‐III cells with 5‐HT7 receptor antagonist administration.

In addition to our current findings, we demonstrated cell viability, apoptosis, and necrosis of the KATO‐III cell line using flow cytometry and the Hoechst 33258 technique. Analysis of the KATO‐III flow cytometry findings revealed a 79.83% decrease in viable KATO‐III cells following administration of the antagonist alone. These results indicate that the 5‐HT7 receptor antagonist reduces proliferation in the KATO‐III cell line. It also repaired impaired apoptosis in KATO‐III cells and induced apoptosis in 14%. The apoptotic pathway, however, did not function effectively with concurrent administration of the 5‐HT7 receptor agonist and 5‐HT7 receptor antagonist. Additionally, Hoechst 33258 staining results showed that the 5‐HT7 receptor antagonist induced the formation of dense chromatin networks. The apoptotic and antiapoptotic gene pool alterations we discovered following RT‐PCR analysis are also supported by the results of apoptosis in flow cytometry and Hoechst 33258 staining that 5‐HT7 receptors in gastric cell lines might have an important role in cancer formation or pathogenesis. In culture studies, the administration of 5‐HT7 receptor agonists further increased both proliferation and receptor expression in the KATO‐III cell line. Antagonist administration also decreased the proliferation of cell lines and positively affected parameters such as NF‐kB, BAX, CAS‐3, and CAS‐9, which are thought to contribute to cancer pathogenesis.

In this study, treatment with the 5‐HT7 receptor antagonist SB‐269970 led, for the first time, to a significant reduction in 5‐HT7 receptor expression in GC and KATO‐III cells, alongside an apoptosis‐inducing effect. As expected, the inhibition of 5‐HT7 receptors with SB‐269970 resulted in a marked decline in cell proliferation in both GC and KATO‐III cells. This outcome is supported by the following observations: (1) mRNA expression of the 5‐HT7 receptor is elevated in GC tissue compared to healthy gastric tissue. (2) The proliferation of GC and KATO‐III cells declines in a time‐dependent manner following SB‐269970 administration. (3) SB‐269970 application increases mRNA expression of apoptosis‐related genes, including CAS‐3, CAS‐9, and BAX. (4) The incidence of apoptosis is elevated in the presence of Annexin V/PI, particularly following the administration of SB‐269970. Additionally, the formation of dense chromatin networks has been observed through the utilization of Hoechst 33342 staining.

This study has two main limitations. First, an increase in gene expression generally indicates a corresponding increase in the end product; however, these results may not accurately reflect the expression of the related signaling proteins. Ideally, we would have demonstrated changes in protein expression, but we were unable to do so due to insufficient protein availability and budget constraints. Second, we have only assessed SB‐269970 as a 5‐HT7 receptor antagonist and LP‐44 as an agonist; many other potential ligands for these receptors warrant further investigation.

## 5 Conclusion

In summary, 5‐HT7 receptor antagonists attenuated cell growth and proliferation in both primary GC cells and the KATO‐III cell line by regulating the apoptotic pathway. Improving the body's anticancer defense mechanisms through regulating BAX, CAS‐3, and CAS‐9 expression by antagonism of 5‐HT7 receptors supports the possible anticancer effect of 5‐HT7 antagonists. However, further in vivo and in vitro studies, as well as clinical investigation, are required to explore its mechanism of action. Future studies should investigate the role of 5‐HT7 receptors in other types of GC and clinical cases, confirming the effectiveness of antagonism of this receptor in cancer treatments. This study suggests that 5‐HT7 receptors should be evaluated as potential targets in oncology, as they may enable new approaches for treating gastric cancer (GC). Additionally, the antagonism of these receptors could be explored as a potential anticancer therapy protocol.

## Author Contributions

Irfan Cinar, Busra Dincer, Elif Cadirci, and Zekai Halici conceived and designed the study. Mehmet Ilhan Yildirgan and Salih Kara surgical tissue collection and preservation. Elif Cadirci, Irfan Cinar, Busra Dincer, and SSPY performed experimental works. Elif Cadirci and Irfan Cinar wrote the draft of the manuscript. Irfan Cinar and Busra Dincer analyzed the data. Irfan Cinar, Elif Cadirci, Zekai Halici, and Busra Dincer researched the data, contributed to the discussion, and reviewed/edited the manuscript. All authors read and approved the final version of the manuscript. Elif Cadirci is the guarantor of this study and, as such, has full access to all the data in the study and takes responsibility for the integrity and accuracy of the data. The authors declare that all data were generated in‐house, and no paper mill was used.

## Ethics Statement

It was approved by the Ataturk University, Faculty of Medicine, Noninvasive Clinic Research Ethical Committee (20.03.2014; 4/11) that all phases of our studies were consistent with the ethical rules.

## Conflicts of Interest

The authors declare no conflicts of interest.

## Data Availability

The data that support the findings of this study are available on request from the corresponding author. The data are not publicly available due to privacy or ethical restrictions.
